# Skin mucus proteins of lumpsucker (*Cyclopterus lumpus*)

**DOI:** 10.1016/j.bbrep.2016.12.016

**Published:** 2017-01-05

**Authors:** Deepti Manjari Patel, Monica.F. Brinchmann

**Affiliations:** Faculty of Biosciences and Aquaculture, Nord University, 8049 Bodø, Norway

**Keywords:** Lumpsucker, *Cyclopterus lumpus*, Skin mucus, 2D gel, Mass spectrometry, Proteome, Mucosal immunity, Stress, Biomarker

## Abstract

Fish skin mucus serves as a first line of defense against pathogens and external stressors. In this study the proteomic profile of lumpsucker skin mucus was characterized using 2D gels coupled with tandem mass spectrometry. Mucosal proteins were identified by homology searches across the databases SwissProt, NCBInr and vertebrate EST. The identified proteins were clustered into ten groups based on their gene ontology biological process in PANTHER (www.patherdb.org). Calmodulin, cystatin-B, histone H2B, peroxiredoxin1, apolipoprotein A1, natterin-2, 14-3-3 protein, alfa enolase, pentraxin, warm temperature acclimation 65 kDa (WAP65kDa) and heat shock proteins were identified. Several of the proteins are known to be involved in immune and/or stress responses. Proteomic profile established in this study could be a benchmark for differential proteomics studies.

## Highlights

• A proteome reference map of lumpsucker skin mucus was established.
• Proteins involved in immune and stress responses were identified in skin mucus of *Cyclopterus lumpus*.
• Mucosal proteins identified could be potential biomarkers.

## 1 Introduction

*Cyclopterus lumpus* L.*,* commonly known as lumpsucker/lumpfish, is a semi-pelagic fish distributed throughout the North Atlantic Ocean. This fish has been valued for its roe in fish food industry for decades [1]. Recently, use of this species as a delousing agent in salmon farms has gained interest. Lumpsucker is found to be a suitable candidate for delousing in waters even at lower temperatures where other cleaner fish might not thrive well [2]. Despite of the advantages of using lumpsucker as a cleaner fish there is a risk of transmission of diseases to the farmed salmon from infected lumpsuckers, needing further studies. Equally important is the understanding and management of the health and welfare of the lumpsucker itself. Bacterial infection is one major constraint in lumpsucker farming. There are several pathogens causing diseases in lumpsucker such as *Pasteurella sp*., atypical *Aeromonas salmonicida, Vibrio anguillarum, V. ordalii, Vibrio sp., Tenacibaculum sp., Paramoeba perurans, Gyrodactylus* sp. Infections were found to be more prevalent when fishes were stressed either by transport, vaccination and/or introduction to new environment [3]. Relatively little is known about lumsucker's biology and immune system, especially at the molecular level.

In fish, skin is one of the major sites for pathogen entry as it is a mucosal surface with living cells throughout. The skin mucus has a very important role in maintaining fish health, especially in intensive farming where level of stress and infections could be high. Skin mucus of fish contains a variety of immune relevant factors including lectins, lysozymes, calmodulin, immunoglobulins, complement, C-reactive proteins, proteolytic enzymes, anti-microbial peptides and proteins [4]. These factors form a biochemical barrier that serves as first line of defense against a wide range of pathogens. Characterization of skin mucus has been approached from different aspects focusing either on a particular protein of interest or a group of proteins. Recent studies use high throughput techniques for skin mucus characterization in fish. These include characterization of the i) proteome reference map of naïve Atlantic cod (*Gadus morhua*) skin mucus [5], ii) differential skin mucus proteome of Atlantic cod upon natural infection with *V. anguillarum* [6], iii) proteomic profile of discus fish (*Symphysodon aequifasciata*) skin mucus showing parental care [7], iv) proteomic profile of gilthead seabream (*Sparus aurata*) skin mucus [8], [9], v) proteomics profile of European sea bass (*Dicentrarchus labrax*) [10], v) changes in protein composition of Atlantic salmon (*Salmo salar*) skin mucus followed by sea lice (*Lepeoptheirus salmonis*) infection [11], vi) skin mucus and sting venom of marine catfish (*Cathorops spixii*) revealing functional diversification of toxins [12].

Here we describe the skin mucus proteome of lumpsucker by using 2D gels coupled with mass spectrometry. We found immune relevant as well as stress physiology relevant proteins. These results could be useful for implementation of health and stress management strategies for production of a more robust fish.

## 2 Materials and methods

### 2.1 Fish and skin mucus sampling

Lumpsucker used in this study were provided by Arctic Cleanerfish, Stamsund, Norway. They were transported as newly hatched larvae, further held at Mørkvedbukta Research Station, Bodø, Norway, where they were start-fed with Gemma Micro and later fed with Amber Neptun of increasing sizes (1–4 mm). Both commercial feeds were from Skretting, Stavanger, Norway. The juveniles were raised on filtered seawater from 250 m depth, at 10–12 °C for the first 60 days and then the temperature was lowered to 7 °C until sampling. One-year-old fishes weighing approximately 700 g of varying length were anesthetized with MS-222 (70 mg/l) and killed by a blow to the head. For sampling of skin mucus the fish was kept on a plastic bag and massaged gently for a few seconds, discarding samples contaminated with feces. The mucus was transferred into tubes with the help of a spatula. The tubes were immediately frozen and stored at −80 °C until further analysis. All animal handling procedures were performed under to the regulations set by National Animal Research Authority in Norway.

### 2.2 Sample preparation for 2-DE

Protein samples from skin mucus of eight fishes were extracted individually. For sample preparation the protocol of Wang et al. [13] was followed with few modifications. In brief, the skin mucus was thawed on ice and diluted with one volume of PBS containing 0.1% protease inhibitor (GE Healthcare, USA). The samples were sonicated (2×5 s) using an ultrasonic processor (SONICS Vibracell VCX750, USA). Next, the sonicated skin mucus was centrifuged at 15,000*g* for 30 min, 4 °C to pellet the tissue debris and the supernatant was collected. A mixture of TCA (trichloroacetic acid), 10% w/v and 0.1% DTT (DL-Dithiothreitol, Sigma, USA) was added to the supernatant and incubated on ice for 30 min. The sample containing TCA and DTT was centrifuged at 10000*g* for 30 min, 4 °C. The pellet was resuspended in cold acetone containing 0.1% DTT and incubated at −20 °C for 45 min. The sample was centrifuged again at 10,000*g* for 30 min, 4 °C, the pellet obtained was air dried for 2–3 min and dissolved in rehydration buffer (9.8 M urea, 2% CHAPS (3-[(3-Cholamidopropyl) dimethylammonio]-1-propanesulfonate), 20 mM DTT, 0.5% Biolyte (3–10), and 0.001% bromophenol blue, all from Sigma, except Biolyte from Bio-rad). The protein sample in rehydration buffer was used for two dimensional gel electrophoresis.

### 2.3 Two-dimensional gel electrophoresis

The protein content was estimated using Qubit® Protein Assay Kit and Qubit™ fluorometer (Life Technologies, USA) following the manufacturer's protocol. 17 cm (pH-3–10), IPG strips (immobilized pH gradient, Bio-Rad, USA) were rehydrated for 15 h using 80 µg of protein per strip. The rehydrated strips were subjected to iso-electric focusing in Bio-Rad Protean IEF cell to a total volt hours of 60,000 at a maximum of 10,000 V using three steps of slow ramping at a constant temperature of 20 °C [5]. The focused IPG strips were reduced with 0.2% DTT and alkylated with 0.3% iodoacetamide for 15 min each in equilibration buffer (6 M urea, VWR; 0.375 M tris-HCl (pH 8.8), Bio-Rad; 2% SDS, 20% glycerol, Sigma). The equilibrated gel strips were loaded on 12.5% polyacrylamide gels in the Bio-Rad Protean IIxii system (USA). Initially, the gels were run at constant current of 20 mA/gel for 15 min and then 6 mA/gel overnight (approx. 16 h). The following day, current was increased to 15 mA/gel to complete the run. The voltage was limited to 250 V throughout the run. The gels were stained with Sypro® Ruby Protein gel stain, Life technologies, USA, following the manufacturers protocol and images were documented using ChemiDoc™ XRS system (Bio-Rad). The documented gel images were analyzed in PDQuest™ Advanced 2D analysis software (Bio-Rad) to identify consistent spots over 6 gels. Fifty spots with high expression levels in the skin mucus of lumpsucker were selected for analysis.

### 2.4 LC-MS/MS

A preparative gel was run with a protein content of 300 µg and stained with Sypro® Ruby as described by Kulkarni et al. [14]. The selected spots from the PDQuest analysis were excised manually on a blue light transilluminator (Safe Imager™ 2.0 Blue- Light Transilluminator, Life technologies, USA). The excised spots were trypsinized, reduced in gel, alkylated and subjected to LC-MS/MS analysis [15]. The analysis was performed with nanoAcquity ultra-performance liquid chromatography and Q-TOF Ultima global mass spectrometer (Micromass/Waters, MA, USA) at University Proteomics Platform, University of Tromsø, Norway.

### 2.5 Protein identification using bioinformatics tools

The LC-MS/MS analysis generated pkl (powered keylogger) files by using the Protein Lynx Global server software (version 2.1, Micromass/Waters, MA, USA). The pkl files obtained were analyzed using MASCOT MS/MS Ions search (version 2.4.01) against SwissProt protein database (10 Jul 2015, 548872 sequences) and NCBI non-redundant database (10 Jul 2015, 69146588 sequences). In places where SwissProt or NCBInr could not identify the protein, search was carried out against vertebrate EST database (10 Jul 2015, 54205008 sequences). The parameters set for protein identification were enzyme trypsin with one missed cleavage, fixed modification carbamidomethyl of cysteine and variable modification oxidation of methionine, peptide charge 2+ and 3+, peptide tolerance 100 ppm and MS/MS ion tolerance 0.1 Da. The search was performed for the taxonomic class, actinopterygii (ray finned fishes). All searches were carried out using the decoy search and the false discovery rate (FDR) were kept below 1% for both peptide matches above identity and homology threshold. Protein hits above significant threshold score and having at least one unique peptide sequence were identified.

### 2.6 Gene ontology (GO) enrichment analysis

For GO enrichment analysis UniProt IDs of identified proteins were retrived from UniProt knowledgebase (UniProtKB). The UniProt IDs were submitted to PANTHER (www.pantherdb.org) to cluster the proteins into different groups relating to their biological process according to gene ontology annotation (GO terms). Only results with p<0.05 were accepted. A protein-protein interaction network with a medium confidence score was created using string v9.05.

## 3 Results and discussion

At present, there are various techniques for mapping the proteome, however classical 2D gels still have their place in the field of protein and molecular biology. Benefits of using 2D gels include direct visualization of proteins giving a scope for assessment of the sample quality, ability to separate proteins even with small changes in pI and molecular weight, hence possibilities for identification of modifications in protein isoforms such as post translational changes or differences resulting from alternatively spliced mRNAs. It also serves as a powerful tool for identification of proteins from organisms with a non-sequenced genome by the help of *de novo* sequencing and homology searches [16].

**Fig. 1:**
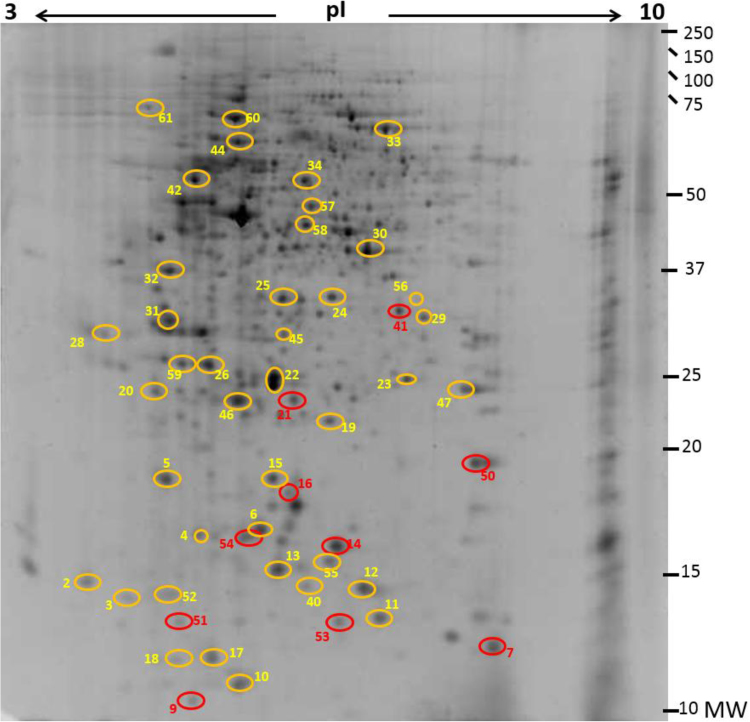
*Cyclopterus lumpus* skin mucus was sonicated, acid precipitated and dissolved in rehydration buffer (Section 2.2) then 80 µg were loaded unto 17 cm, 3–10 non-linear IPG strips. 12.5% polyacrylamide vertical gels were used as the second dimension. The image shows a representative gel with the spots analyzed with mass spectrometry circled. Yellow rings represent identified spots, red rings; not identified spots.

In this study, proteins from naïve lumpsucker skin mucus were identified using 2D gels coupled with LC-MS/MS. Skin mucus proteins (100 μg/strip) from eight fishes were electro focused and ran on 12.5% polyacrylamide gels. A representative gel image is shown in Fig. 1. Out of ≈900 spots detected by PDQuest, only fifty highly expressed spots were excised for LC-MS/MS analysis but 40 spots were possible to identify using database searches. To our knowledge this is the first report on the skin mucus proteome of lumpsucker, *C. lumpus*. Lumpsucker's genome has not been sequenced and very little information on the species is available in the databases. Thus, the proteins were identified adapting homology searches restricting the BLAST searches to the class Actinopterygii (ray finned fishes). Details of individual proteins are listed in Table 1.

**Table 1:** MASCOT analysis details, gene symbols and physical parameters of identified protein spots from lumpsucker skin mucus.

### 3.1 Immune and stress related proteins in skin mucus of lumpsucker

**Fig. 2:**
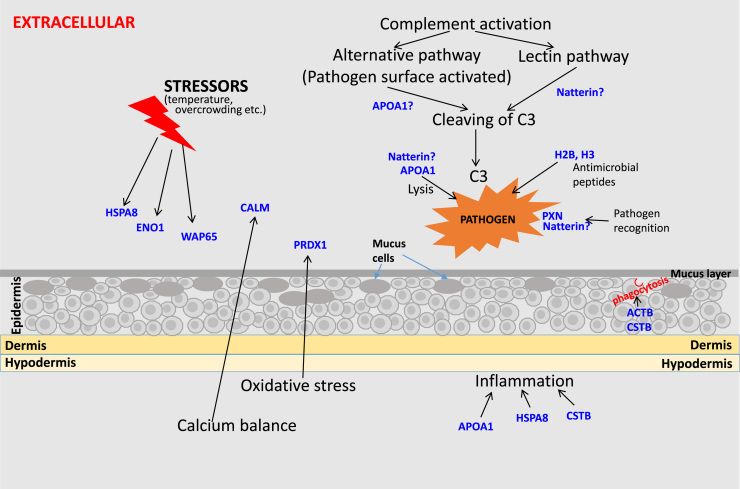
Possible interactions of some of the identified proteins from lumpsucker skin mucus are shown. Proteins in the figure are indicated by their abbreviations. Arrows indicates their involvement in different process. Question mark “?” indicates proposed actions of the proteins. Biological roles of the proteins are explained in text in results and discussion section.

In this study spot 19 was identified as peroxiredoxin 1 (PRDX1). It has also been reported in skin mucus of naïve gilthead seabream (*S. aurata*) [8] and European seabass (*Dicentrarchus labrax*) [10]. Peroxiredoxins, also known as thioredoxin peroxidase are cysteine-based peroxidases grouped as 1-cys or 2-cys according to the number of their cysteine-conserved residues [17]. These are antioxidant proteins that protect the organism from toxic reactive oxygen species (ROS) during oxidative stress (Fig. 2). It also participates in various biological processes such as molecular chaperoning, hydrogen peroxide mediated cell signaling and mitochondrial functions. PRDX1 is also called natural killer enhancing factor A, has been implicated in immune responses of many organisms. In fish the relatively high expression level of PRDX1 in immune related tissues like spleen and kidney of golden pompano (*Trachinotus ovatus*) suggests its role in immunity of this species [18]. In infection studies, the expression of PRDX1 was downregulated in *Neoparamoeba perurans* infected *S. salar* [19] and *Enteromyxum leei* infected *S. aurata* [20]. Phagocytic cell produces ROS to eliminate pathogens. Hence, downregulation of the PRDX1 gene may facilitate phagocytosis for removal of pathogens. Further, it has been reported that extracellular peroxiredoxin 1 could act as endogenous danger signal by binding to cell membrane sensors or receptors [21].

Lectins are specific carbohydrate binding proteins involved in a variety of biological roles. Here we identified two lectins namely natterin (spot 22) and pentraxin (spot 46). Natterin was first isolated from venom gland of *Thalassophryne natteri* [22] Natterin like proteinaceous toxins (I and II) were purified from skin secretions of oriental catfish (*Plotosus lineatus*) [23]. Natterin has a pore forming toxin like domain with kinogenase activity [22]. The lectin like domain in natterin is homologous to Jacalin domain identified in jack fruit. Little work has been done on natterin in fish but jacalin, the plant homologue, is reported to be involved in activation of human T- lymphocytes [24] and apoptosis of B-lymphocytes [25] suggesting a role in the immune system of fish. In mucus the lectin domain could give direct interaction with pathogens and the pore forming toxin domain could potentially result in lysis (Fig. 2).

Pentraxins, spot 46, are evolutionarily conserved proteins with a variety of roles in host defense. As acute phase proteins, their role in inflammatory responses and pathogen recognition make them important markers of infection and inflammation (Fig. 2) [26]. Pentraxin is found in skin mucus of common skate [27], surprisingly the skin gene expression was not changed after in vivo challenge with *E.coli.* This might suggest that the skin and mucus levels of pentraxin are constant, or that this particular pathogen does not stimulate pentraxin production in skin, but it does not exclude that proteins synthesis in liver where human pentraxins are produced could change. Further studies are needed to investigate mucus pentraxin function.

Spot 2 was identified as calmodulin. Previously calmodulin was identified in skin mucus of sea lice infected Atlantic salmon [11] and *Vibrio anguillarum* infected Atlantic cod [6]. This protein was also purified from skin mucus of tilapia (*Sarotherodon mossambicus*), and identified in mucus from European sea bass (*Dicentrarchus labrax*) [10]. Calmodulin is calcium binding multifunctional protein highly conserved in all eukaryotes. It is involved in cell signaling, stress and immune responses. Calmodulin is an important calcium binding protein found to be highly expressed in Antarctic notothenioid fishes when compared to warm water fish, this could indicate a protective role against cold stress [28]. Further, studies reported that over expression of the Antarctic notothenioid calmodulin gene in transgenic tobacco plants showed an increase in cold tolerance when grown at 4 °C for two weeks [29]. In chinese mitten crab (*Eriocheir sinensis*) [30] and blue mussel [31] the calmodulin gene was significantly upregulated in gills and hepatopancreas under salinity and pH stress. This indicates that calmodulin might help to combat stress. Calmodulin could also have role in immune responses against pathogens. Silencing of calmodulin gene in *Penaeus monodon* made it susceptible to *Vibrio harveyi* infection resulting in increased mortality. This could be that silencing of calmodulin gene decreases the transcription of other immune related proteins required for the initiation of immune cascade [32]. Upregulation of this gene was reported in gills of chinese mitten crab challenged with *Edwardsiella tarda* and *V. anguillarum* [30], and in hemocytes of Pacific white shrimp infected by *V. parahemolyticus* [33]. Thus calmodulin in lumpsucker skin mucus might be involved in transduction of signals for downstream immune responses.

We also identified histone proteins, histone H2B (Spot 3) and histone H3 (Spot 13). Histones are major component of the nucleosomes and well known for their role in gene transcription regulation in eukaryotic cells. Studies have shown that there are also extranuclear histones present in mitochondria and on cell surfaces, with many physiologically important roles [34]. Histones released to the extracellular space serve as danger associated molecular patterns. Histones also serve as antimicrobial peptides that could either kill the pathogens directly or indirectly by blocking the DNA/RNA/protein synthesis (Fig. 2) [35], [36]. H2B like protein isolated from skin mucus of Atlantic cod showed antimicrobial activity against *E. coli[* [37]. Similarly H2B like protein in skin of Channel catfish (*Ictalurus punctatus)* showed antimicrobial activities against *Aeromonas hydrophila* and *Seprolegnia* spp. [38]. Further studies indicated that the level of histone like proteins were suppressed in channel catfish exposed to stress [39]. Histones are also identified in skin mucus of naïve European seabass [10].

Cystatin-B, also known as stefin-B, is a protease inhibitor, which regulates the activities of cysteine proteases. This protein is involved in both physiological and pathological conditions such as inflammatory responses (Fig. 2), protein homeostasis, antigen processing and metastasis. Spot 11 was identified as cystatin-B like protein. The presence of cystatin B in mucus might give protection against invading pathogen by inhibiting the cysteine proteases released from pathogens to promote their growth and proliferation. A protease inhibitor from epidermis of Japanese eel (*Anguilla japonica)* has been found to inhibit the proteolytic activity of cysteine proteases of *Porphyromonas gingivalis* [40]. Significant changes in cystatin-B level was observed in Atlantic salmon infected by *Neoparamoeba perurans* [41], and in turbot (*Scopthalmus maximus)* infected by *Ectalurus tarda* [42]. In *S. maximus* cystatin-B were also involved in bacterial invasion of head kidney macrophages [42]. This protein has also been identified in skin mucus of Atlantic cod [5].

Apolipoprotein A1 (spot 20) is a major component of high-density lipoprotein in plasma mainly involved in lipid metabolism [43]. It also plays anti-inflammatory role in both acute and chronic inflammation [44]. This protein was upregulated in skin mucus of sea lice infected Atlantic salmon [11], *Vibrio anguillarum* infected Atlantic cod [6]. Furthermore, in channel catfish this protein also showed lytic activities against Gram positive *Micrococcus lysodeikticus* and Gram negative *Aeromonas hydrophila* [45]. The carboxyl end of this protein is responsible for antimicrobial activities that might give protection against pathogens in skin mucus of teleost fish (Fig. 2) [46]. Apolipoprotein A1 has also been identified in skin mucus of naïve European sea bass [5] and Atlantic cod [10].

Warm temperature acclimation protein 65 kDa (WAP65) is homologous to mammalian hemopexin, a glycoprotein involved in transportation of heme from site of hemolysis. It could protect the skin against bacterial invasion by limiting available iron essential for bacterial proliferation and establishment. In this study spot 64 was identified as WAP65. Upregulation of WAP65 was observed in copper treated swordtail fish, *Xiphophorus helleri* [47] and upregulation of hemopexin like protein mRNA found in hypoxia induced longjaw mudsucker, *Gillichthys mirabilis* [48]. Differential expression of WAP65 was also observed due to warm temperature and bacterial infections in channel catfish [49]. Goldfish WAP65 contains a cytokine response element, suggesting a role in self-defense [50]. In naïve European sea bass WAP65 is present in skin mucus [10].

Heat shock proteins are highly conserved proteins involved in various stress responses including heat, heavy metal exposure, tissue damage, and pathogen infections (Fig. 2). These are molecular chaperones that helps the organism to repair the protein damage occurred due to adverse stress conditions. Spot 60 and 61 were identified as a heat shock 70 kDa protein and heat shock cognate 71 kDa protein respectively. Heat shock proteins exists both intracellularly and extracellularly. Extracellular HSPs have been reported to act as immune modulators, that could be immunostimulatory or immunosuppressive depending on how they are encountered by the immune response network [51]. Heat shock protein 70 has been found in skin mucus of European sea bass [10] and gilthead seabream [8].

Enolases are a glycolytic enzyme, which also acts as plasminogen receptor, transcriptional regulator and cell associated stress protein (Fig. 2) [52]. Spot L34 was identified as alfa enolase in lumpsucker skin mucus. Alfa enolase serves as a stress marker in fish showing upregulation during hypoxic conditions in longjaw mudsucker (*Gillicthys mirabilis*) [48]. Studies also showed upregulation of alfa enolase in *Sparus aurata* after *in vivo* LPS challenge [52].

The protein 14-3-3 was identified from four spots (26, 28, 32, 59) with varying pI and molecular weight suggesting several isoforms in skin mucus of lumpsucker. These are highly conserved proteins found ubiquitously in animal tissues. They are signaling proteins associated with osmoregulatory signal transduction in *Fundulus heteroclitus* gill epithelium [53]. This protein has also been involved in phagocytosis and microbial resistance in zebrafish. Knock down of this gene in zebrafish infected with *Staphyloccocus aureus* showed decrease in survival rate than control fish indicates its role in bacterial resistance [54].

### 3.2 Other identified proteins

We also identified cytoskeletal proteins such as actin (spots 45, 57 and 58), Septin-2 (Spot 58), keratin (spots 33 and 24), F-actin capping protein (spot 25), myosin (spot 5). Actin is a dynamic protein that plays several roles in the cell. It is found to be involved in cell movement, phagocytosis (Fig. 2), cytokinesis and cytoplasmic streaming. Previously actin fragments have been identified in skin mucus of sea lice (*Lepeophtheirus salmonis*) infected Atlantic salmon (*Salmo salar*). Some of the proteins identified in lumpsucker skin mucus are enzymes involved in various metabolic pathways i.e. nucleoside diphosphate kinase B (spots 12 and 40), triosephosphate isomerase B (spot 23), glyceraldehyde 3-phosphate dehydrogenase (spot 30), malate dehydrogenase (spot 56) and ATP synthase (spot 42).

Identification of the proteins in skin mucus indicates a role in the extracellular space. Several delivery routes could be used to reach the outside of the cell [61]. That might be i) secreted through the ER- Golgi classical pathway, ii) released to the extracellular space by exosomes, iii) released by necrotic cells, iv) released from the endolysosomal pathway or v) by some unknown pathway yet to be discovered. Table 2 gives an overview of the identified proteins and of their previously known presence in extracellular space and/or skin mucus of fish.

**Table 2:** GO biological process of identified proteins.

Spot ID	Protein name	Biological process										Reported in skin mucus	Present extracellularly
		B1	B2	B3	B4	B5	B6	B7	B8	B9	B10		
L2	Calmodulin				✓							Y [6]	Y [55]
L3	Histone H2B 1/2			✓	✓				✓			Y	Y [56]
L4	Predicted: Lipocalin-like				✓			✓	✓			–	Y [57]
L5	Myosin, light polypeptide 9, like 1					✓				✓		–	–
L6	Growth/differentiation factor 6-A	✓	✓		✓	✓			✓		✓	–	Y
L10, L17,L18	Glial fibrillary acidic protein			✓	✓	✓						–	–
L11	Predicted: Cystatin-B-like		✓						✓			Y [5]	–
L12, L40	Nucleoside diphosphate kinase B	✓	✓		✓				✓			Y [5], [8], [9]	Y
L13	Histone H3.2			✓					✓			–	Y [56]
L15	60 S ribosomal protein L11								✓			Y [8]	Y
L19	Peroxiredoxin 1						✓		✓			Y [8]	Y [58]
L20	Predicted: apolipoprotein A-I-like		✓		✓	✓		✓	✓	✓	✓	Y [8], [9]	Y
L22	Natterin-2											–	Y
L23	Triosephosphate isomerase B								✓			Y [5], [8], [9]	Y
L24, L33	Keratin, type I cytoskeletal 13			✓	✓	✓						Y [8]	Y
L25	Predicted: F-actin-capping protein subunit beta isoforms 1 and 2-like isoform X1		✓	✓	✓	✓			✓			–	–
L26, L28, L32, L59	14-3-3 protein beta/alpha				✓							Y [5], [8]	Y [59]
L29	Guanine nucleotide-binding protein subunit beta-2-like 1				✓			✓				–	–
L30	Glyceraldehyde 3-phosphate dehydrogenase isoform 2								✓			Y [5], [8], [9]	–
L31	Charged multivesicular body protein 4c							✓				–	Y
L34	Alpha-enolase								✓			Y [5], [8]	Y
L42	ATP synthase subunit beta, mitochondrial							✓	✓			Y [8], [9]	Y
L44	Warm-temperature-acclimation-related 65 kDa protein							✓				Y [8], [9]	Y
L45	Actin, cytoplasmic			✓	✓			✓				Y [5], [9]	Y
L46	Predicted: pentraxin fusion protein-like						✓				✓	Y [27]	Y [27]
L47	Glutathione-S-transferase											Y [5], [9]	Y
L52	Coactosin-like protein			✓	✓							Y [8], [9]	Y
L55	DNA-binding protein RFX2		✓		✓				✓			–	–
L56	Malate dehydrogenase 2-2, NAD								✓			Y [9]	Y
L58	Predicted: septin-2				✓				✓			–	–
L60	Heat shock 70 kDa protein 8b1			✓			✓		✓		✓	Y [7], [9]	Y [60]
L61	Heat shock cognate 71 kDa			✓			✓		✓		✓	Y [9]	Y [60]

B1; apoptotic process (GO:0006915), B2; biological regulation (GO:0065007), B3; cellular component organization or biogenesis (GO:0071840), B4; cellular process (GO:0009987), B5; developmental process (GO:0032502), B6; immune system process (GO:0002376), B7; locali

zation (GO:0051179), B8; metabolic process (GO:0008152), B9; multicellular organismal process (GO:0032501), B10; response to stimulus (GO:0050896).

“Y” means yes, the protein has been identified in skin mucus of fish or its extracellular presence has been observed. Information is based on UNiProtKB in places where references are not cited.

### 3.3 Gene ontology analysis

**Fig. 3:**
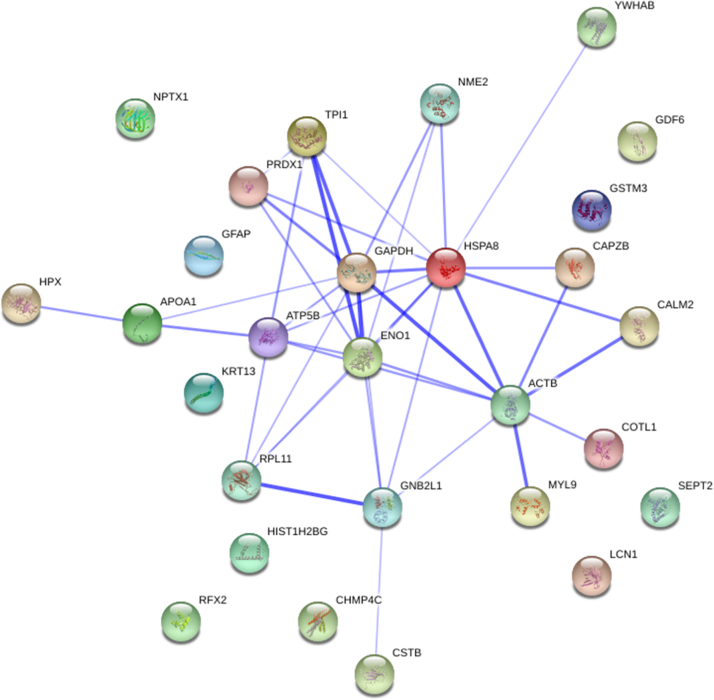
Confidence view of protein-protein interaction network of identified proteins created by string v9.05. Bolder lines mean higher confidence.

The gene IDs for the 40 identified spots were obtained from UniProtKB for GO analysis. Gene IDs for all identified proteins could not be obtained for the fish model organism, zebrafish. Hence, the IDs used here were the human orthologs of the respective proteins identified in lumpsucker skin mucus except natterin-2, which do not have a human ortholog in UniProtKB. The GO biological process clustered the proteins into ten groups (Table 2) such as apoptotic process (GO:0006915), biological regulation (GO:0065007), cellular component organization or biogenesis (GO:0071840), cellular process (GO:0009987), developmental process (GO:0032502), immune system process (GO:0002376), localization (GO:0051179), metabolic process (GO:0008152), multicellular organismal process (GO:0032501) and response to stimulus (GO:0050896). The GO biological process indicated the involvement of individual proteins in several processes, which are listed in Table 2. A confidence view (medium confidence score) protein-protein interaction network was created using String v9.05 employing the human UniProt IDs (Fig. 3). The interaction results need to be studied in an extracellular setting such as mucus, to establish if functional protein interaction network exist in mucus alone or in mucus interacting with skin cells and/or pathogens.

## 4 Conclusion

This study revealed the presence of several proteins that are involved in immune and stress responses in skin mucus of lumpsucker. Some of these proteins could be potential biomarkers for fish welfare. Thus, the proteome reference map of lumpsucker skin mucus could serve as a benchmark for future studies on lumpsucker, although this needs to be verified by additional research.
