# Supplementary material for: Skin mucus proteins of lumpsucker (Cyclopterus lumpus)
Source: Biochem Biophys Rep. 2017 Jan 5;9:217–25. doi: 10.1016/j.bbrep.2016.12.016 (PMC5614610; doi:10.1016/j.bbrep.2016.12.016)
Supplement: Transparency document [file mmc1.docx]

Dear Editor,	Bodø, Norway 25 August 2016

Regarding the submitted manuscript “Skin mucus proteins of lumpsucker (Cyclopterus lumpus)” the authors declare that there are no conflict of interest.

Yours faithfully,

Monica F. Brinchmann.
